# Malnutrition and Its Influence on Sepsis Outcomes in Elderly Patients

**DOI:** 10.7759/cureus.63433

**Published:** 2024-06-29

**Authors:** Muhammad Daud, Fahim Ullah, Muhammad Uzair, Ambar Siddiq, Urooj Siddiq, Fahad Bin Riaz, Musawer Ibrar, Ayesha Hamid Khan

**Affiliations:** 1 General Surgery, Lady Reading Hospital, Peshawar, PAK; 2 Internal Medicine, Lady Reading Hospital, Peshawar, PAK; 3 Medicine, Médecins du Monde, Peshawar, PAK; 4 Surgery, Lady Reading Hospital, Peshawar, PAK; 5 Medicine, Nowshera Medical College, Nowshera, PAK

**Keywords:** complications, mortality, elderly, sepsis, malnutrition

## Abstract

Background: Malnutrition is common among the elderly and has significant implications for hospitalization outcomes. This is particularly true for severe illnesses such as sepsis, given age-related physiological changes and comorbidities.

Objective: This study examined how malnutrition affected hospitalization outcomes in older adults admitted for sepsis.

Materials & methods: A prospective cohort study was conducted at Lady Reading Hospital in Peshawar, Pakistan, from January to December 2023, focusing on 390 sepsis patients aged 55 years and older. Data on clinical history, length of stay, mortality rates, comorbidities, and nutritional assessments were collected using standardized forms. After controlling for confounding variables, statistical analysis using SPSS version 23 (IBM Corp., Armonk, NY) examined the relationship between hospitalization outcomes and nutritional status.

Results: The research included 390 elderly sepsis patients and showed significant variations in the demographics, comorbidities, and severity of disease between the well-nourished and malnourished groups. Malnourished patients had higher rates of ICU admission (52.82% vs. 29.23%), mechanical ventilation (45.13% vs. 16.41%), mortality (27.18% vs. 14.87%), and 30-day readmission (28.21% vs. 12.82%) as compared to the well-nourished group. They also had longer hospital stays (18.1 days vs. 12.6 days). Malnutrition significantly influenced outcomes, with multivariate analysis indicating it as a predictor of longer stays (β = 2.8, p < 0.001) and increased mortality risk (OR = 3.2, 95% CI: 1.9-5.4, p < 0.001).

Conclusion: Malnutrition significantly worsens outcomes for elderly sepsis patients, increasing ICU admissions, ventilation needs, mortality rates, and readmissions, emphasizing the need for proactive nutritional interventions.

## Introduction

Malnutrition is a major worldwide health problem, particularly among older adults. Its impact on hospitalization outcomes, particularly for severe illnesses like sepsis, is significant [1,2]. Due to age-related physiological changes and comorbidities, older adults face substantial challenges with sepsis, a potentially fatal condition caused by an excessive immune response to infection [3,4]. Despite advancements in medical treatment, sepsis remains a leading cause of morbidity and mortality in the elderly worldwide [5].

Numerous studies have explored the link between malnutrition and adverse health outcomes in the elderly [6]. Malnutrition, defined as the body's inability to absorb necessary nutrients or to use them efficiently, impairs immunological function, slows the healing of wounds, and leads to frailty, all conditions that might make sepsis more likely to occur [7,8]. Additionally, malnourished older adults often have low energy levels, which may make it harder for them to fight off serious diseases [9].

Optimizing clinical care for older persons with sepsis requires an understanding of the complex interactions between hospitalization outcomes and malnutrition [10,11]. Although previous studies have shown a link between undernourishment and unfavorable consequences in a range of medical ailments, there is still a significant void in the literature about its precise influence on sepsis outcomes in the elderly [12]. This gap underscores the need for comprehensive studies that delve into the intricate pathways linking malnutrition to sepsis-related morbidity and mortality in this vulnerable population.

This study aims to investigate the effects of malnutrition on hospitalization outcomes in elderly patients treated for sepsis, thereby addressing this research gap. Our goal is to shed light on the relationships that exist between clinical features, sepsis outcomes, and nutritional status to provide useful insights that may guide focused treatments and enhance the care of older adults with sepsis.

Research objective

This study examines how malnutrition affects hospitalization outcomes in older adults admitted for sepsis.

## Materials and methods

Study design and settings

The Lady Reading Hospital (LRH) in Peshawar, Pakistan, served as the research site for this prospective cohort. The research was conducted from January to December 2023, allowing for comprehensive data collection and analysis.

Inclusion and exclusion criteria

The trial included patients aged 55 years or older, diagnosed with sepsis according to the standard clinical criteria (including evidence of systemic inflammatory response syndrome (SIRS) in the presence of infection), and hospitalized at LRH. Patients with pre-existing malnutrition-related diseases, such as advanced cancer cachexia, and those transferred from other hospital institutions with inadequate clinical information were among the exclusion criteria. By excluding potential confounding variables such as pre-existing malnutrition-related diseases (e.g., advanced cancer cachexia) and patients transferred from other hospital institutions with inadequate clinical information, this strategy aimed to ensure the research sample's homogeneity. This focus allowed for a more precise evaluation of the impact of malnutrition on hospitalization outcomes for sepsis in older adults.

Sample size

The technique for calculating proportions was used to calculate the sample size of 390 participants with a margin of error of 5% and a confidence level of 95%, taking into account the expected prevalence of malnutrition among older persons with sepsis.

Data collection

Data were collected prospectively from eligible patients using standardized data collection forms. Documentation encompassed clinical background, length of stay, mortality, complications, comorbidities, nutritional status (anthropometric measurements and laboratory parameters), illness severity scores (Acute Physiology and Chronic Health Evaluation II (APACHE II) and Sequential Organ Failure Assessment (SOFA)), and nutritional interventions during hospitalization.

Statistical analysis

SPSS version 23 (IBM Corp., Armonk, NY) was used for statistical analysis. The research population's clinical and demographic features were compiled using descriptive statistics. Categorical data were shown as frequencies and percentages, whereas continuous variables were given as means ± standard deviations or medians with interquartile ranges. The independent t-test was used to compare the means of continuous variables (length of stay). The chi-square test was used to compare the proportions of categorical variables (ICU admission rate, mechanical ventilation, mortality rate, readmission rate, acute kidney injury, respiratory failure, septic shock, delirium, and pressure ulcers). To calculate the p-values for comparing the means of each characteristic between the malnourished and well-nourished groups, we used an independent t-test. P-value <0.05 was significant.

Ethical approval

The Institutional Review Board (IRB) of Lady Reading Hospital, Medical Teaching Institution (MTI) issued approval for the current study. Before beginning the study, all participants or their legal representatives gave their informed permission, and patient anonymity was scrupulously maintained at all times.

## Results

The mean age of the malnourished group (n = 195) was 64.1 years (SD ± 8.4), comprising 93 females (47.69%) and 102 males (52.31%). As far as education goes, out of the total number of patients, 84 (43.08%) had completed high school, 66 (33.85%) had completed college, 26 (13.33%) had completed university education, and 19 (9.74%) were illiterate (Table 1). Marital status was as follows: 45.64% were married, 37.44% were widowed, 12.31% were single, and 4.62% were divorced. Furthermore, of the patients, 133 (68.21%) were retired, 19 (9.74%) were working, and 43 (22.05%) were unemployed. In a similar vein, the mean age of the well-nourished group (n = 195) was 62.7 years (SD ± 8.0), including 88 female patients (45.13%) and 107 male patients (54.87%). Regarding education, out of the patients, 63 (32.31%) had completed high school, 74 (37.95%) had completed college, 33 (16.92%) had completed university education, and 25 (12.82%) were illiterate. There were 109 married patients (55.90%), 46 widowed patients (23.59%), 27 single patients (13.85%), and 13 divorced patients (6.67%). There were 122 patients (62.56%) who were retired, 39 patients (20.00%) who were working, and 34 patients (17.44%) who were unemployed.

**Table 1:** Demographic characteristics of malnourished and well-nourished elderly sepsis patients.

Characteristic		Malnourished (n = 195)		Well-nourished (n = 195)	
		n	%	n	%
Age (years)	(Mean ± SD)	64.1 ± 8.4		62.7 ± 8.0	
Gender	Male	102	52.31	107	54.87
	Female	93	47.69	88	45.13
Education level	School	84	43.08	63	32.31
	College	66	33.85	74	37.95
	University	26	13.33	33	16.92
	Illiterate	19	9.74	25	12.82
Marital status	Married	89	45.64	109	55.90
	Widowed	73	37.44	46	23.59
	Single	24	12.31	27	13.85
	Divorced	9	4.62	13	6.67
Employment status	Retired	133	68.21	122	62.56
	Employed	19	9.74	39	20.00
	Unemployed	43	22.05	34	17.44

The clinical features and nutritional status evaluations of 195 undernourished and 195 well-nourished sepsis patients are shown in Table 2. The following are the mean ± standard deviation values for several parameters: BMI (kg/m²) of the malnourished group was 21.5 ± 2.8, while the BMI of the well-nourished group was 24.7 ± 3.6; the albumin level (g/dL) was 2.5 ± 0.5 in the malnourished group and 3.3 ± 0.6 in the well-nourished group; the pre-albumin level (mg/dL) was 16.2 ± 4.7 in the malnourished group and 21.2 ± 5.2 in the well-nourished group; the hemoglobin (g/dL) was 10.5 ± 1.8 in the malnourished group and 11.9 ± 2.0 in the well-nourished group; the serum creatinine (mg/dL) was 1.4 ± 0.5 in the malnourished group and 1.1 ± 0.2 in the well-nourished group; and the glucose (mg/dL) was 130 ± 25 in the malnourished group and 110 ± 15 in the well-nourished group. The p-values (<0.001) indicate that the differences in BMI, albumin level, pre-albumin level, hemoglobin, serum creatinine, and glucose between the malnourished and well-nourished groups are statistically significant.

**Table 2:** Clinical and nutritional status of elderly sepsis patients. * P-value < 0.05 is considered significant.

Characteristic	Malnourished (mean ± SD)	Well-nourished (mean ± SD)	t-value	P-value*
BMI (kg/m²)	21.5 ± 2.8	24.7 ± 3.6	-9.80	<0.001
Albumin level (g/dL)	2.5 ± 0.5	3.3 ± 0.6	-14.30	<0.001
Pre-albumin level (mg/dL)	16.2 ± 4.7	21.2 ± 5.2	-9.96	<0.001
Hemoglobin (g/dL)	10.5 ± 1.8	11.9 ± 2.0	-7.27	<0.001
Serum creatinine (mg/dL)	1.4 ± 0.5	1.1 ± 0.2	7.78	<0.001
Glucose (mg/dL)	130 ± 25	110 ± 15	9.58	<0.001

The comorbidities and severity of disease ratings of 195 patients with malnutrition and 195 patients with adequate nutrition who were hospitalized for sepsis are shown in Table 3. In the malnourished group, 56 (28.72%) had chronic obstructive pulmonary disease (COPD), 40 (20.51%) had cardiac disease, 28 (14.36%) had renal disease, and 98 (50.26%) had hypertension. By contrast, 83 patients (42.56%) with hypertension, 52 patients (26.67%) with diabetes, 29 patients (14.87%) with COPD, 35 patients (17.95%) with cardiac disease, and 18 patients (9.23%) with renal disease belonged to the well-nourished group. Furthermore, the mean ± standard deviation values for the SOFA score were 8.3 ± 3.3 and 10.1 ± 3.6, respectively, and for the APACHE II score, they were 24.5 ± 6.1 in the malnourished group and 21.0 ± 6.2 in the well-nourished group.

**Table 3:** Comorbidities and severity of illness in malnourished vs. well-nourished elderly sepsis patients. APACHE II: Acute Physiology and Chronic Health Evaluation II; SOFA: Sequential Organ Failure Assessment.

Characteristic	Malnourished (n = 195)		Well-nourished (n = 195)	
	n	%	n	%
Hypertension (mmHg)	98	50.26	83	42.56
Diabetes (mg/dL)	81	41.54	52	26.67
Chronic obstructive pulmonary disease	56	28.72	29	14.87
Heart disease	40	20.51	35	17.95
Renal disease	28	14.36	18	9.23
APACHE II score (mean ± SD)	24.5 ± 6.1		21.0 ± 6.2	
SOFA score (mean ± SD)	10.1 ± 3.6		8.3 ± 3.3	

Based on their nutritional state, Table 4 shows the hospitalization results for 390 older patients who were hospitalized with sepsis. The results include the number of days spent in the ICU, the rate of admission, the need for mechanical ventilation, the death rate, and the rate of readmission within 30 days. Among malnourished patients, 103 (52.82%) required ICU admission, 88 (45.13%) required mechanical ventilation, 53 (27.18%) died, and 55 (28.21%) were readmitted within 30 days. Their mean stay was 18.1 days (SD ± 6.2). On the other hand, patients who were well-nourished had a shorter average duration of stay, measuring 12.6 days (SD ± 4.7). They also had reduced rates of death (14.87%), mechanical ventilation (16.41%), ICU admission (29.23%), and 30-day readmission (12.82%).

**Table 4:** Hospitalization outcomes based on nutritional status in elderly sepsis patients.

Outcome	Malnourished (n = 195)	Well-nourished (n = 195)	Total (N = 390)
Length of stay in days (mean ± SD)	18.1 ± 6.2	12.6 ± 4.7	15.3 ± 5.8
ICU admission rate	103 (52.82%)	57 (29.23%)	160 (41.03%)
Mechanical ventilation	88 (45.13%)	32 (16.41%)	120 (30.77%)
Mortality rate	53 (27.18%)	29 (14.87%)	82 (21.03%)
Readmission rate (30 days)	55 (28.21%)	25 (12.82%)	80 (20.51%)

**Figure 1:**
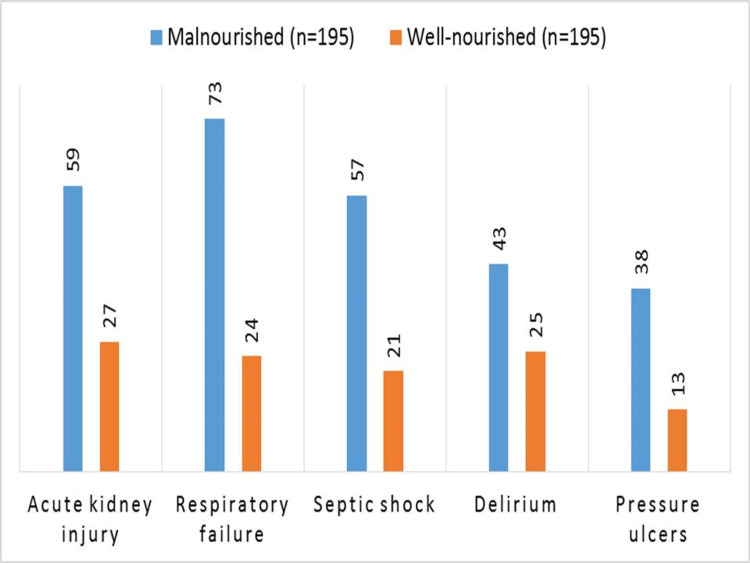
Hospitalization complications in malnourished vs. well-nourished elderly sepsis patients.

According to their nutritional condition, Figure 1 shows the problems that were seen during hospitalization among 390 older persons who were treated for sepsis. Acute renal damage, respiratory failure, septic shock, disorientation, and pressure ulcers are potential complications. Acute renal damage occurred in 59 instances, respiratory failure in 73 cases, septic shock in 57 cases, delirium in 43 cases, and pressure ulcers in 38 cases among malnourished patients. In contrast, there were 27 cases of acute renal damage, 24 cases of respiratory failure, 21 cases of septic shock, 25 cases of delirium, and 13 cases of pressure ulcers among patients who were well-nourished.

**Figure 2:**
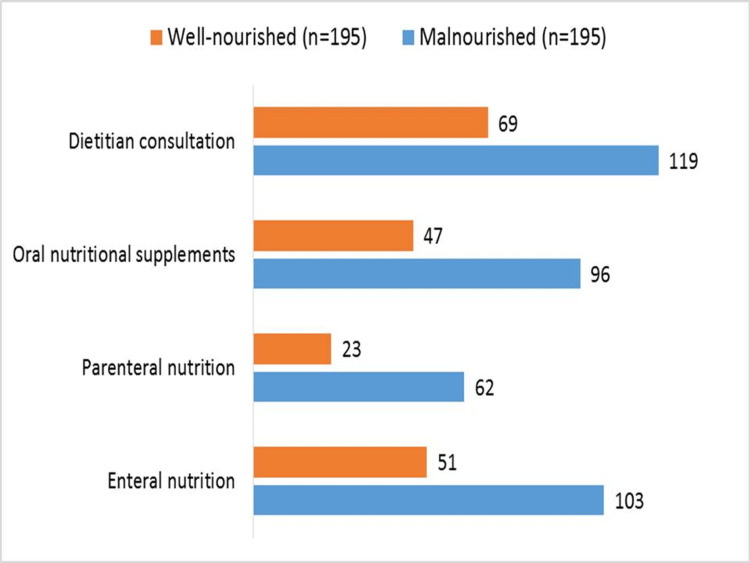
Nutritional interventions for elderly sepsis patients during hospitalization.

According to their nutritional state, Figure 2 shows the dietary therapies given to 390 older persons hospitalized for sepsis. Dietitian advice, oral nutritional supplements, parenteral nutrition, and enteral nutrition are among the therapies. Of the malnourished patients, 96 got nutritional supplements orally, 119 had dietician consultation, 103 underwent enteral nutrition, and 62 underwent parenteral nutrition. On the other hand, the corresponding figures for patients who were well-nourished were 51, 23, 47, and 69, respectively. These approaches emphasize the proactive approach to addressing malnutrition in older persons with sepsis and attempt to meet patients' nutritional requirements throughout hospital stays.

Table 5 provides a thorough summary of the bivariate analysis and illustrates the significant disparities in hospitalization outcomes between older persons who are malnourished and those who are well-nourished and have been treated for sepsis. The mean duration of stay for the 195 malnourished patients was 18.1 days ± 6.2, which was substantially longer than the 12.6 days ± 4.7 for the well-nourished group (p < 0.001). Moreover, there were statistically significant (p < 0.001) increases in the rates of ICU admission (52.82% vs. 29.23%), mechanical ventilation (45.13% vs. 16.41%), death (27.18% vs. 14.87%), and readmission within 30 days (28.21% vs. 12.82%) among malnourished persons. Additionally, compared to the well-nourished group, the malnourished patients had higher frequencies of delirium (22.05% vs. 12.82%), respiratory failure (37.44% vs. 12.31%), septic shock (29.23% vs. 10.77%), pressure ulcers (19.49% vs. 6.67%), and acute kidney injury (30.26% vs. 13.85%). All of these differences were statistically significant (p < 0.001).

**Table 5:** Analysis of nutritional status and hospitalization outcomes in elderly sepsis patients. * Independent t-test; ** chi-square test.

Outcome	Malnourished		Well-nourished		p-value
	n	%	n	%	
Length of stay (days)	18.1 ± 6.2		12.6 ± 4.7		<0.001*
ICU admission rate	103	52.82	57	29.23	<0.001**
Mechanical ventilation	88	45.13	32	16.41	<0.001**
Mortality rate	53	27.18	29	14.87	<0.001**
Readmission rate (30 days)	55	28.21	25	12.82	<0.001**
Acute kidney injury	59	30.26	27	13.85	<0.001**
Respiratory failure	73	37.44	24	`12.31	<0.001**
Septic shock	57	29.23	21	10.77	<0.001**
Delirium	43	22.05	25	12.82	<0.001**
Pressure ulcers	38	19.49	13	6.67	<0.001**

The findings of the multivariate analysis that looked at variables related to the duration of stay for older persons hospitalized with sepsis are shown in Table 6. A noteworthy positive correlation was found between malnutrition and length of stay (β = 2.8, standard error (SE) = 0.6, p < 0.001), suggesting that patients with malnutrition spent more time in the hospital on average than those with adequate nutrition. Higher APACHE II score (β = 1.5, SE = 0.3, p < 0.001), higher SOFA score (β = 1.2, SE = 0.3, p < 0.001), older age (β = 0.2, SE = 0.1, p = 0.02), and the presence of comorbidities (β = 1.0, SE = 0.4, p = 0.01) were other significant predictors of longer duration of stay. On the other hand, a shorter length of stay was linked to greater albumin levels (β = -0.8, SE = 0.2, p < 0.001), indicating that improved nutritional condition, as indicated by albumin levels, can potentially shorten hospital stays.

**Table 6:** Multivariate analysis of factors affecting length of stay in elderly sepsis patients. P-value < 0.05 is considered significant. APACHE II: Acute Physiology and Chronic Health Evaluation II; SOFA: Sequential Organ Failure Assessment.

Variable	Beta (β)	Standard error (SE)	p-value
Malnutrition	2.8	0.6	<0.001
Age	0.2	0.1	0.02
APACHE II score	1.5	0.3	<0.001
SOFA score	1.2	0.3	<0.001
Comorbidities	1.0	0.4	0.01
Albumin level	-0.8	0.2	<0.001

The findings of a multivariate study looking at variables linked to death among older individuals hospitalized for sepsis are shown in Table 7. Significant correlations between a number of factors and death rates are found in the investigation. A significant risk factor was found to be malnutrition, with an odds ratio of 3.2 (95% CI: 1.9-5.4, p < 0.001), meaning that those who were malnourished had a 3.2 times higher chance of dying than those who were well-nourished. A slight but statistically significant correlation between aging and mortality was also seen, with an odds ratio of 1.1 (95% CI: 1.0 - 1.3, p = 0.04). Increased mortality risk was also highly connected with higher scores on the severity of illness evaluations, such as SOFA scores (odds ratio: 1.6, 95% CI: 1.2-2.1, p < 0.001) and APACHE II (odds ratio: 1.8, 95% CI: 1.4-2.3, p < 0.001). Additionally, a greater chance of death was linked to the existence of comorbidities, with an odds ratio of 1.5 (95% CI: 1.1-2.0, p = 0.01).

**Table 7:** Multivariate analysis of mortality risk factors in elderly sepsis patients. APACHE II: Acute Physiology and Chronic Health Evaluation II; SOFA: Sequential Organ Failure Assessment.

Variable	Odds ratio (OR)	95% confidence interval (CI)	p-value
Malnutrition	3.2	1.9-5.4	<0.001
Age	1.1	1.0-1.3	0.04
APACHE II score	1.8	1.4-2.3	<0.001
SOFA score	1.6	1.2-2.1	<0.001
Comorbidities	1.5	1.1-2.0	0.01

## Discussion

Our study examined the complex association between hospitalization outcomes and malnutrition in older patients suffering from sepsis, a condition known for its severe consequences. Notable differences were found between the cohorts of malnourished and well-nourished individuals based on the demographic study. In contrast, well-nourished patients had a slightly lower mean age of 62.7 years (SD ± 8.0) and a comparable gender distribution. Malnourished patients had a mean age of 64.1 years (SD ± 8.4), with 52.31% of them being male. These results are consistent with other research showing that elderly patients with comorbidities have a greater frequency of malnutrition [13,14].

According to our research, patients who were malnourished had greater incidences of chronic illnesses than patients who were well-nourished. In particular, COPD accounted for 28.72% of malnourished patients, cardiac disease for 20.51%, renal disease for 14.36%, and hypertension for 50.26% [15,16]. On the other hand, those who were well-nourished had reduced rates of these chronic conditions: 14.87% had COPD, 17.95% had heart disease, 9.23% had renal disease, and 42.56% had hypertension [17]. These results support earlier studies showing that a number of comorbidities are often associated with malnutrition, which may worsen sepsis and lead to unfavorable consequences [18].

Notably, hospitalization outcomes for elderly sepsis patients were significantly influenced by their nutritional state, including longer stays, higher ICU admission rates, increased mechanical ventilation needs, elevated mortality rates, and higher readmission rates within 30 days. Those who were underweight spent an average of 18.1 days (SD ± 6.2) in the hospital, whereas those who were well-nourished spent 12.6 days (SD ± 4.7). This conclusion is consistent with other research that found a link between extended hospital stays in critically sick patients and malnutrition [19,20]. The fact that malnourished patients remain longer indicates how important it is to provide focused nutritional therapies to speed up recovery and reduce medical expenses.

In elderly patients with sepsis, malnutrition was linked to an increased rate of admissions to the intensive care unit (ICU) and the need for mechanical ventilation. In particular, compared to the well-nourished group, which had rates of 16.41% and 29.23% for mechanical ventilation and ICU hospitalization, respectively, malnourished persons had far higher rates of both. These results are consistent with other studies that showed a similar trend of higher ICU admissions and mechanical ventilation among patients with nutritional sepsis [21]. To reduce the risk of respiratory issues and enhance overall outcomes, this vulnerable group has an increased requirement for critical care treatments, which highlights the urgent need for proactive nutritional assistance.

Our study found a statistically significant difference in the death rates of elderly individuals with sepsis who were either malnourished or well-nourished. The death rate for malnourished persons was 27.18%, a significant increase from the 14.87% recorded for the well-nourished group. These results are in line with other studies that found a substantial correlation between malnutrition and a higher risk of death in sepsis patients [22]. The increased risk of death highlights the importance of nutritional optimization in managing sepsis in older individuals to improve survival rates. This study's strength lies in its comprehensive analysis of the impact of malnutrition on hospitalization outcomes in elderly sepsis patients through a large, well-defined cohort and robust statistical methods. The increased risk of death underscores the critical importance of nutritional optimization as a key component in managing sepsis in older individuals to improve survival rates. However, the limitation of this study is its single-center design, which may limit the generalizability of the findings to other settings or populations.

## Conclusions

Our study underscores the critical relationship between malnutrition and hospitalization outcomes in elderly patients with sepsis. Malnourished individuals experienced prolonged hospital stays, higher rates of ICU admission, mechanical ventilation, readmission, and mortality compared to their well-nourished counterparts. Moreover, malnutrition was associated with a greater burden of comorbidities and severity of illness, exacerbating the risk of adverse outcomes. These findings emphasize the urgent need for proactive nutritional interventions in the management of elderly sepsis patients to improve clinical outcomes and reduce healthcare burden. Addressing malnutrition as an integral part of sepsis care may lead to better survival rates and enhanced quality of life for this vulnerable population.
